# Brief Sketch of the Life and Services of John Hunter

**Published:** 1841-07

**Authors:** 


### REVIEWS.

---

Art. I.—*Brief Sketch of the Life and Services of John Hunter:* Condensed from his Biography by Drewry Ottley. London.

The lives of eminent Medical men constitute important epochs in the history of Medicine. The physician possessed of a proper degree of professional pride should not be ignorant of Medical biography, for although it may be admitted that, in a practical point of view, such knowledge is of secondary importance, yet we regard it essential to an accomplished Medical education.

That it is a neglected branch cannot be denied. Every practitioner of medicine has doubtless heard of Hippocrates, Galen, Sydenham, Cullen, or Rush; but how many can give any thing approaching an accurate account of their lives and services? Alas! Not one in fifty.

This ignorance, we think, is inexcusable. The literature of our profession is a deeply interesting and useful study, and we know that it is easy of acquisition. Further, we cannot doubt that a more extended acquaintance with Medical literature would serve to improve the profession both in a social and scientific aspect. One of the greatest hindrances to the elevation of our character as a body, is that sectional prejudice, which we regret to say, too often developes itself in the deportment and writings of the leading members of the profession. This is decidedly injurious. One common interest should bind us together, be we residents of Maine or Louisiana, London, Paris, or Calcutta; and among the means calculated to preserve our unity, that of which we are speaking stands pre-eminent.

We propose giving a brief account of the life and services of one of the most distinguished medical philosophers of modern times, and we shall be gratified if our effort shall incite any one to a more thorough inquiry.

John Hunter was born at Long Calderwood, a short distance from Glasgow, on the 14th of February, 1728. At the age of ten he lost his father, and having a very indulgent mother, was permitted to engage, without restraint, in all the frivolities of boyhood. His early education was neglected. Indeed it seems that Hunter, in his younger days, had little or no inclination to confine his attention to books. He was fond of sporting in all its varieties, and of anything in which he could display manual dexterity. It was a source of great regret to Mr. Hunter in his after life, as well as to his many friends, that he had neglected instruction in his tender years; and it is certain, that nothing but the force of insuppressible genius aided by a lofty and an honorable ambition, could have sustained him under the many difficulties which he necessarily encountered.

At the age of twenty he left his native place and repaired to London, where he put himself under the care of his brother, Dr. William Hunter, whose well earned fame was spreading far and wide, and whose high standing seemed to give a new and vigorous impulse to the mind of John.

After entering upon his duties as a pupil, "no long time elapsed before his skill was put to the test in preparing for the lecture a dissection of the muscles of the arm. It is probable that William Hunter had not as yet formed a very high estimate of his hitherto idle brother, and little foresaw that he was ere long to eclipse his preceptor. He was, however, so well pleased with his pupil's first essay that he soon after intrusted him with a similar part, of which the bloodvessels were injected. In this the young student again succeeded so well as to obtain much praise for his dexterity from his brother, who foretold that he would soon become a good anatomist, and promised that he should never want employment. From this time, therefore, we may consider Hunter as engaged in the dissecting room, under the instruction of his brother's assistant, Mr. Symonds, where he pursued his studies with such zeal and diligence, that by the next session he was able to take charge of directing the pupils in their dissections."

About eight months after his arrival in London, by the exertion of his brother William, he gained admittance as a pupil at the Chelsea Hospital, under the celebrated Cheselden, who at that time was perhaps the first surgeon in the world. Here he attended during the summer months of 1749–50, while his winters were occupied in dissecting for his brother. In 1751 Cheselden resigned his office in consequence of bad health, and Hunter entered at St. Bartholomews under the direction of the distinguished Pott. Here he remained but a short time, but was regular in his attendance, and doubtless imbibed many useful principles from his great instructor.

At the death of Cheselden, in 1752, Pott soon placed himself at the head of the Surgical profession in Great Britain. He was the first surgeon in the United Kingdom who renounced the old absurdities of the art, and based his practice upon anatomical facts and physiological principles. But his student was destined to eclipse him, as will appear in the sequel.

In 1754, Hunter, having determined to devote himself to surgery, entered as surgeon's pupil at St. George's Hospital, where he attended regularly in the summer, while, as usual, he devoted himself closely during the winter months to his dissections. Two years after his entrance here, he served in the capacity of House surgeon, to the entire satisfaction of the attending surgeons, and to his own great advantage. "Hunter's extensive acquaintance with anatomy was now generally known and appreciated, and he was not unfrequently applied to by others to assist them in clearing up some difficulties they might encounter in their researches. It was on an occasion of this kind, and about this time, that the important discovery of the mode of connexion between the placenta and the uterus was made. The honor of solving this anatomical problem was laid claim to by each of the Hunters, and the dispute to which it gave rise, twenty years afterwards, caused a breach between them that was never healed until William was on his death bed, and scarcely it would seem in his mind even then."

Hunter in this year became connected with his brother in his anatomical school, and delivered a part of the lectures. As a dissector, he was, without dispute, superior to his brother, but as a lecturer William was infinitely superior to John. In truth John Hunter never became popular as a lecturer. His style was course, and his manner uncouth; and it is a remarkable fact that, notwithstanding the eminence to which he attained in his profession, he never attracted a large audience to his lecture room. Among his earliest contributions to anatomy and physiology, were dissections, observations and experiments, illustrating the office of the lymphatics—the descent of the testis in the fœtus, and the ramification of the olfactory nerves upon the lining membrane of the nose. These, together with a great number of beautiful anatomical specimens, were made between the years 1754–59, after which, his health becoming impaired, he applied for an appointment in the army, and was accordingly made Staff surgeon.

"Notwithstanding his constant employment in the practical duties of his profession whilst in the army, Hunter found time to pursue those physiological researches in which he took supreme delight. He made several experiments on lizards and snakes to ascertain whether digestion continues during their torpid state; and he was also engaged in some inquiries on the faculty of hearing in fishes. It was during these campaigns too, that most of his observations on gun shot wounds were made, and that many of the peculiar views which his work on inflammation unfolds, first suggested themselves to his mind, though they were not published until more than thirty years afterwards, as he constantly aimed, during his whole life, to confirm and amend them, and to build them up into a work on which his future fame should depend."

"In 1763, peace having been proclaimed, Hunter accompanied the forces home, and on arriving in England returned to the metropolis."

We come now to an important era in the life of this distinguished man—a period when his talents and fortitude were called in daily exercise in order to sustain him under many trying difficulties. Many circumstances conspired to render it necessary for him to undergo a tedious probation. His income was narrow, and he was obliged to live in rather a retired manner. A number of able surgeons already occupied the field, among whom were Pott, Bromfield, Sir Cæsar Hawkins, Sharp and Warner. Hunter's manners were any thing but prepossessing; in truth, he was rude, undignified, and what was still worse, addicted to profanity. Add to these his deficient elementary education, and we may readily conjecture what was the fact—his slow progress to celebrity. But he was unceasing in his devotions to his favorite pursuits. He was not content with resting upon the knowledge of the anatomists and surgeons who preceded him. His restless spirit and searching intellect sought for a better exposition of the curious operations of the animal fabric than had ever been given. Accordingly we find him engaged in the study of comparative anatomy and physiology, morbid anatomy and surgery.

Shortly after his location in London, he commenced delivering lectures on surgery and anatomy, which he continued for several years. It is remarkable, however, that his auditors never amounted to twenty. But he was not to be discouraged at the sight of empty benches. His zeal increased with obstacles. The charnel house was his home. He cared not for lecture room eloquence, or for elegant acquirements of any kind whatever. He had projected a most magnificent scheme, and he was resolved to devote himself unremittingly to its consummation. This scheme was no other than the formation of the vast museum which now bears his name, and which, without dispute, is superior to any collection of the kind in the world. His time and money were completely invested in the enterprize. It was the darling object of his head and heart, and the records of science no where exhibit such an instance of enthusiasm as was displayed in the prosecution of this gigantic work. "Sir Everard Home used to state, that as soon as he accumulated fees to the amount of ten guineas, he always purchased some addition to his collection. Indeed he was not unfrequently obliged to borrow of his friends when his own funds were at a low ebb and the temptation was strong. "Pray George," said he one day to Mr. G. Nicol, "have you got any money in your pocket?" Mr. N. replied in the affirmative. "Have you got five guineas? because if you have and will lend it to me, you shall go halves." "Halves in what?" inquired his friend. "Why halves in a magnificent tiger which is now dying in Castle street." Mr. Nicol lent the money and Hunter got the tiger."

In addition to his labors in Zoology, Hunter was sedulously engaged in surgical and pathological researches, and without detriment to other men, we may truly affirm that he is to be regarded as the father of modern surgery. All who are acquainted with his work on the blood, and his views upon inflammation, will at once see the justice of this remark.

In 1767 he was elected a fellow of the Royal Society. In the same year he ruptured his tendo-achillis, and the circumstance afforded him an opportunity for investigating the manner in which divided tendons unite. He cut the same tendon in dogs, and found that union took place as in fractured bones where the skin is not wounded.*

In 1768, at the age of 40, he was elected surgeon of St. George's Hospital, and shortly afterwards he was chosen a member of the Corporation of Surgeons. "By his election to

*It is singular that Hunter should not have devised the operation of Tenotomy for Club-Foot and other distortions. He was certainly the first surgeon who fully demonstrated the fact, that divided tendons will reunite, (a point which had been long denied,) but it was not until seventeen years after Hunter's experiments that Tenotomy was undertaken.

the hospital he was ensured the means of making his talents as a surgeon more generally known, and was enabled to obtain as private pupils, on advantageous terms, young gentlemen coming to town to complete their medical education. * * * Amongst those who successively became inmates of Hunter's house, as private pupils, were Dr. Jenner, Mr. Guy, Mr. Kingston, Dr. Physick, and Sir Everard Home."

In May, 1771, he published the first part of his work on the Teeth, containing a description of those organs in their natural state, and in 177– he published the second part, giving an account of their diseases. In this year ('71) he was married to Miss Home, Sir Everard's sister, to whom he had been engaged a number of years, and with her he lived pleasantly until his death notwithstanding their habits were somewhat uncongenial.

In 1772, five years after his election as a fellow of the Royal Society, Hunter presented, for the first time, a paper before this body on the digestion of the stomach after death, which was published in the Philosophical Transactions. Shortly after this he presented another paper to the same body giving "an account of the electric organs of the Ray, and pointed out the vast nerves with which they are supplied, as the probable source of their peculiar power."

In the autumn of '72, he delivered a course of lectures on the principles of surgery. We have before observed that Hunter was a poor speaker. He had an aversion to public speaking, insomuch that he was in the habit of taking laudanum a short time before his lecture hour. "His language, though forcible, was inelegant and often coarse; his delivery was heavy and unengaging, as he rarely raised his eyes from his book; and as, in addition to this, the doctrines he taught were new and often obscure and theoretical, his hearers were never numerous."

As an instance of his coarseness, it is related by his biographer (Ottley,) that in speaking of a case of gun-shot wound, he described the ball as "having gone into the man's belly and hit his guts such a d—d thump that they mortified."

Hunter's philosophy, as exhibited in his lectures and papers for the Royal Society, was strictly Baconian. He paid little or no regard to mere opinions. He had little respect for speculations and theories no matter by whom projected. His book knowledge was limited. He observed closely, and drew all his inferences from visible, tangible facts. He never allowed himself to rest contented with a superficial examination of any subject. His productions always evinced depth and comprehension of research. Originality was displayed in every sentence he wrote, in every lecture he delivered.

"Hunter's increasing professional avocations began to render it impossible for him to devote as much time as he wished to extending and perfecting his collection. The field of his labors too was considerably increased on the one hand, by his opportunities for the cultivation of morbid anatomy, which his connexion with a hospital and his increasing private practice afforded him; on the other, by the augmented stores of rare and curious specimens of the animal kingdom which the kindness of his friends, and the liberality of scientific men, continually accumulated on his hands." He was, therefore, under the necessity of engaging a gentleman to assist him in his labors. Notwithstanding this he was incessantly devoted to his studies and dissections.

"He commenced his labors in the dissecting-room generally before six in the morning, and remained there until nine, when he breakfasted. After breakfast he saw patients at his own house until twelve, when he made it a point to set forth on his rounds, even though persons might be in waiting for the purpose of seeing him. He dined at four and was a very moderate eater. After dinner he was accustomed to sleep for about an hour, and his evenings were spent either in preparing or delivering lectures, in dictating to an amanuensis the records of particular cases of which he kept a regular entry, or in a similar manner committing to paper the substance of any work on which he chanced to be engaged." He continued his labors until one or two o'clock in the morning, long after his family had retired.

In the early part of '75, Hunter presented an able paper to the Royal Society, on animal and vegetable heat, demonstrating "that living bodies possess a power of maintaining their heat against the influence of external cold, and this in proportion to their rank in the scale of organization."

In 1776 he was appointed Surgeon Extraordinary to the King. In the same year, at the solicitation of the Humane Society, he prepared an essay on the means to be employed in the resuscitation of drowned persons.

"He also commenced this year a series of six Croonian lectures on muscular motion, which he had been appointed by the Royal Society to deliver."

About this time he commenced a correspondence with Jenner, who had been one of his favorite pupils. Jenner had settled at Berkley, where he made the splendid discovery which has conferred upon his name an enviable immortality. He took great delight in assisting his illustrious preceptor in the prosecution of his labors, in return for which, Hunter gave him his advice in relation to any difficult case on which he might be consulted.

The following letter will afford an example of Hunter's epistolary style of writing, as well as the blunt, coarse manner he had of delivering his opinions.

"Dear Jenner:—You must think me very fond of fish, when you send me cheese as much fishified as possible; however, it is an excellent cheese and every country has laid claim to its birth.

"I have but one order to send you, and that is, to send me every thing you can get, either animal, vegetable, or mineral, and the compound of the two, either animal or vegetable, mineralized.

"I would have you do nothing with the boy but dress him superficially; these funguses will die and be damned to them, and drop off.

"Have you large trees of different kinds that you can make free with? If you have, I will put you upon a set of experiments with regard to the heat of vegetables.

"Have you any eaves where bats go to at night? If you have, I will put you upon a set of experiments concerning the heat of them at different seasons. I should have been extremely happy to have had a visit from Lord Berkley.

"Ever Yours, JOHN HUNTER."

This correspondence with Jenner was continued until the close of the year 1790, shortly before Hunter's death. It related almost entirely to enquiries concerning the habits of a number of animals, such as the cuckoo, the bat, the hedgehog and the porpoise; and in ascertaining their instincts and customs Jenner was of great service to his old preceptor.

Perhaps the most interesting experiments in which Jenner took part, were those relating to animal heat. Hunter's several papers on this subject were very valuable. The following letter relative to this topic, evinces an ardent desire for accurate knowledge, together with a restless spirit, which could only be satisfied by the information afforded him by his friend and pupil.

"Dear Jenner:—* * * * I have received the hedgehogs. If you have time, see their natural winter haunts, and in very cold weather run the thermometer into the anus, and observe the heat; then open the belly by a small hole, and pass the thermometer down towards the pelvis, and observe the heat; then towards the liver and diaphragm and observe the heat: you may do all this in a very few minutes. Observe the fluidity of the blood, by comparing it with another that has been kept warm for a few days. * * * * * I have seen your old master who has given me the use of a very curious bone. I hope he will give it me altogether.

"Dear Jenner, yours,

"*Nov.* 23. JOHN HUNTER."

In 1779 Hunter presented his celebrated paper to the Royal Society, on the hermaphrodite of black cattle, or the free martin, containing not only a description of these singular animals, but observations on hermaphroism in general. In the same year he presented two other papers; one containing an account of the transmission of small pox from a mother to her fœtus in utero; the other on a peculiarity of the hen pheasant, the change of her plumage to the color of the cocks after the cessation of breeding.

In 1781 he was elected a member of the Royal Society of Belles Lettres at Gottenburg.

"In 1782 he completed his series of six Croonian lectures on muscular motion, in which he unfolded many novel and ingenious views respecting the causes of motion in vegetables and animals, and on the various modes of progression employed by the latter in swimming, flying, leaping, running, &c."

In 1783 he was elected a member of the Royal Society of Medicine and the Royal Academy of Surgery of Paris. The same year he built his museum, which cost him about fifteen thousand dollars. This sum, however, is small in comparison to the cost of his specimens which at one time were estimated by Hunter himself at *three hundred and fifty thousand dollars!* During the same year he also assisted in forming the "*Society for the improvement of Medical and Chirurgical Knowledge*," which continued in existence about twenty years, and numbered among its members some of the brightest ornaments of the British profession. Before this society Mr. Hunter read a number of papers which were characterized by great research and originality of thought. Among others we may mention his "observations on the inflammation of the internal coats of veins;" his papers on "Introsusception," and on "Aneurism," and his "Experiments and Observations on the growth of Bones."

Hunter was the author of the operation for the cure of aneurism, which consists in tying the artery at a distance from the tumor, between it and the heart; and notwithstanding many efforts have been made to award the merit of this device to French surgeons, we believe it is now universally conceded that all the credit is justly due to Hunter.

"In 1786, in consequence of the death of Mr. Middleton, Hunter was appointed Deputy Surgeon General to the army. In the early part of the same year, he published his work on the *Venereal Disease*, which had been long expected by the public, and which met with a rapid sale. On this work Hunter had bestowed great pains, and before publication he submitted every part of it to a committee of his friends, consisting of Sir Gilbert Blane, Dr. Fordzee, Dr. David Pitcairn, and Dr. Marshall." This work of our distinguished surgeon is still held in high estimation, and with the annotations of George G. Babington, surgeon of St. George's Hospital, forms, perhaps, the best text-book on the subject at the present day.

"Towards the end of this year he published also his work on the Animal Economy, consisting chiefly of a collection of his most important papers in the Philosophical Transactions, to some of which he has made considerable additions.

In 1787 Hunter presented two papers to the Royal Society; one designed to prove that the wolf, jackal and dog, are animals of the same species; the other upon the anatomy and physiology of the whale, upon which he had spent much time and money.

The ardent desire of Hunter to obtain rare specimens was well exemplified at the time of Obrien's death. This famous Irish giant had been for a long time in a declining state of health, and Hunter sent his man, Houison, to watch for his exit and, if possible, obtain his body. Obrien heard of their manœuvering and left strict orders to have his body watched until a leaden coffin could be made, in which the corpse was to be enclosed, and the whole sunk in the sea. Accordingly upon his death watchers were set, but Hunter himself succeeded in bribing them by paying the sum of *five hundred pounds*. The skeleton of Obrien now stands at the head of the osteological division of the Hunterian Museum.

"The Royal Society this year ('87) conferred on Hunter the Copley medal, as an honorable testimony to the importance of his discoveries in Natural History. He also received, at the same time, another mark of the estimation in which his labors were held by being elected a member of the American Philosophical Society.

Upon the death of Pott in December, 1788, Hunter was, by universal consent, placed at the head of the surgical profession in England. His health had been delicate for some four or five years. He suffered at times with a singular affection of the heart and arteries, attended with syncope and spasmodic twitchings of the muscles of the face, arms, chest, and stomach. This contributed to the aggravation of a naturally irritable temper, until at length it became so indomitable that, when under a paroxysm, he was utterly unfit for any social or professional duty.

"In 1792 Hunter contributed his last paper to the Philosophical Transactions. This contained the results of his observations on the hive bee, continued, with various interruptions, during a period of twenty years."

Shortly after this he transferred his lectureship to his brother-in-law, Sir Everard Home. To him also he committed his manuscripts which it appears were destroyed, and in all probability by the hand of this gentleman, who, doubtless, after committing extensive plagiarism upon the works of his great master and brother-in-law, endeavored to conceal his crime by demolishing the original. After this transfer, Hunter commenced the publication of his work on Inflammation and Gun Shot Wounds, which did not get through the press until after his death. Commendation of this work is unnecessary at this late day. The dissemination of the views contained therein created a new epoch in Surgery, and from that day to the present, this branch of our profession has made a steady and rapid advancement.

In the early part of '93 some difficulty occurred between Mr. Hunter and his colleagues of St. George's Hospital, respecting the fees of pupils and their admittance into the institution. On the 10th of October the board of Governors met to take the matter into consideration, when Hunter appeared before them laboring under great mental agitation. "In the course of his remarks he made some observations which one of his colleagues thought it necessary instantly and flatly to contradict. Hunter immediately ceased speaking, retired from the table, and struggling to suppress the tumult of his passion, hurried instantly to the adjoining room, which he had scarcely reached, when, with a deep groan, he fell lifeless into the arms of Dr. Robertson, one of the physicians of the hospital who chanced to be present." This put an end to the meeting, the board immediately passing a resolution to defer the matter for future consideration.

"The body was examined after death, when the viscera of the belly and head were found loaded with blood, but otherwise in a natural state, with the exception of the carotid arteries and their branches within the skull, which were in parts thickened and ossified. In the chest the left lung had become attached to the costal pleura by old and firm adhesions; but the heart was found to be the chief seat of disease. The pericardium was unusually thickened, but did not contain much fluid. The heart itself was small, appearing too little for the cavity in which it was contained; its diminished size being the result of wasting, and not of strong contraction of its fibres. Two opake white spots were seen on the left auricle and ventricle. The muscular structure of the organ was pale and loose in its texture. The coronary arteries had their branches, which ramify through the heart, converted into long tubes, with difficulty divisible by the knife. The mitral valves were much ossified. The aorta was somewhat dilated, its valves thickened and wanting pliancy, and the inner surface of the artery was studded with opake and elevated white spots.

"Hunter's body was interred in a private manner, in the church of St. Martin-in-the-Fields, accompanied by a few of his medical friends." He died in his sixty-fifth year.

"In person he was about the middle stature, of a vigorous and robust frame, and free from corpulency; his shoulders were high and his neck short. His features were rather large and strongly marked, his eyebrows projecting, his eye of a light color, his cheeks high, and his mouth somewhat underhung. In dress he was plain and gentlemanlike; and his hair, which in youth was of a reddish color, and in his latter years white, he wore curled behind."

In his will Hunter expressed a desire that his Museum should be offered first to the British government, and in case they should refuse to purchase it, he directed it to be sold to a foreign state, as his executors might deem advisable.

After several ineffectual attempts by a number of influential persons to lay the subject before Parliament, it was at length brought before that body in June, 1796, when they agreed to appropriate 15,000*l* for its purchase; a paltry sum indeed for such a magnifiicent collection. The whole was then offered to the College of Physicians, but they declined accepting it, and it was then accepted by the Corporation of Surgeons on very favorable terms.

This museum contained, at the death of Hunter, some 15,000 specimens in anatomy, human, comparative, and morbid; arranged in excellent order, so as to illustrate the science of life, and the body in a state of disease. It has been considerably increased since it passed into the hands of the College of Surgeons, and it will stand in the metropolis of England a monument of the mighty genius and industry of its illusirious founder. W. J. B.

*Cincinnati*, 1841.

